# Giant Ascending Aortic Aneurysm Revealed by Chronic Cough: A Case Report

**DOI:** 10.7759/cureus.89964

**Published:** 2025-08-13

**Authors:** Thomas Neff, Arnaud Robert, Caroline Amicone, Patrick M Honoré

**Affiliations:** 1 Intensive Care Unit, CHU UCL Namur - Godinne, Yvoir, BEL; 2 Intensive Care Unit, CHU Liège, Liège, BEL

**Keywords:** cerebral ischemic lesions, chronic cough, dyspnea, giant ascending aortic aneurysm, severe tracheal compression

## Abstract

Ascending aortic aneurysms are potentially life-threatening conditions that often present with nonspecific symptoms. Giant aneurysms, defined by a diameter exceeding 10 cm, are rare and may lead to compressive symptoms. We report an unusual case of a giant ascending aortic aneurysm discovered during the evaluation of a chronic cough.

We present the case of a 67-year-old woman who presented with a two-year history of persistent dry cough and exertional dyspnea. Physical examination revealed a diastolic murmur, and imaging studies identified a 10.2 × 10 cm ascending aortic aneurysm extending to the arch. Preoperative planning included femoral cannulation for extracorporeal circulation prior to anesthetic induction due to tracheal compression. Surgical replacement of the ascending aorta and arch was performed. Postoperatively, the patient developed a right hemiparesis and homonymous hemianopia due to ischemic cerebral lesions.

This case highlights the diagnostic challenge posed by nonspecific respiratory symptoms in thoracic aortic aneurysms and the importance of early imaging. Airway compression by large aneurysms, though rare, should be considered in chronic cough of unclear etiology.

Clinicians should maintain a high index of suspicion for mediastinal causes in a persistent, unexplained cough. Early use of chest CT can lead to the timely diagnosis of potentially fatal conditions such as giant thoracic aortic aneurysms. Optimal surgical planning and multidisciplinary management are essential to improve outcomes.

## Introduction

Ascending aortic aneurysm is a potentially life-threatening condition, often diagnosed based on nonspecific clinical signs. Its incidental discovery during the workup of chronic cough remains exceptional, although tracheal compressions secondary to large aneurysms are well described in the literature [1,2,3]. Giant aneurysms, defined by a diameter greater than 10 cm, are particularly rare and carry a high risk of complications, including rupture, dissection, or compression of adjacent structures [4,5]. We report the case of a 67-year-old woman in whom a giant ascending aortic aneurysm was diagnosed during the evaluation of a persistent chronic cough, initially misattributed to infection after several years of diagnostic wandering.

## Case presentation

A 67-year-old woman with no significant past medical history other than a remote history of smoking cessation (15 years prior) presented with a chronic dry cough persisting for over two years. The cough was non-productive, without hemoptysis, and associated with moderate exertional dyspnea. There were no chest pains or signs of infection. Several empirical treatments for upper respiratory tract infections had been prescribed without lasting improvement. On physical examination during pulmonary consultation, a grade 3/6 aortic regurgitation murmur was noted at the aortic area, with otherwise unremarkable pulmonary auscultation. Pulmonary function tests showed a mild obstructive ventilatory defect with an FEV1 at 75% of predicted.

**Figure 1:**
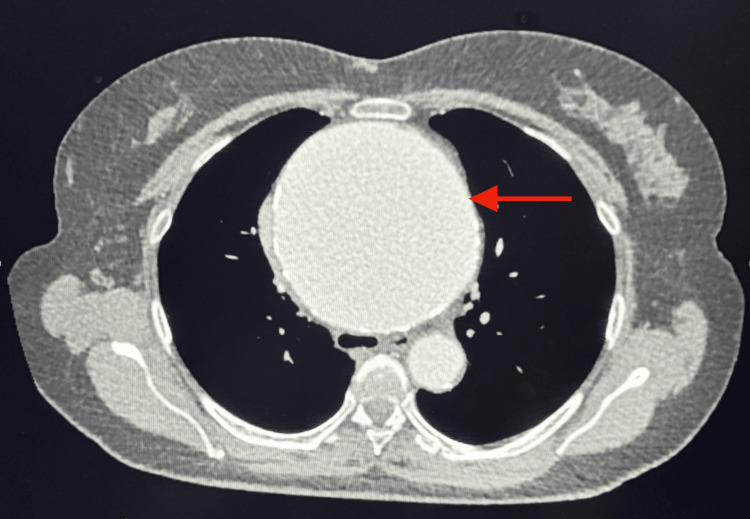
Transverse section of a thoracic CT scan demonstrating a giant ascending aortic aneurysm (red arrow).

In this context, a thoracoabdominal CT scan was performed, revealing a large ascending aortic aneurysm measuring 10.2 × 10 cm, extending up to the aortic arch over a length of 13 cm (Figure 1). No signs of dissection, rupture, or mural hematoma were observed.

Transthoracic echocardiography revealed moderate aortic regurgitation (grade 2/4) without other abnormalities. Preoperative coronary angiography was unremarkable.

Given the high risk of major complications, urgent surgical intervention was scheduled. A median sternotomy was performed, preceded by percutaneous cannulation of the right femoral artery and vein under local anesthesia to establish cardiopulmonary bypass (CPB). General anesthesia was induced only after CPB initiation, due to concerns about difficult intubation and impossible ventilation caused by tracheal compression.

After induction, the patient experienced desaturation, prompting a bronchoscopic examination, which revealed collapse of the left main bronchus, confirming tracheobronchial compression by the aneurysm. The procedure involved replacement of the ascending aorta and aortic arch with a vascular graft, including reimplantation of the brachiocephalic trunk and the left common carotid artery. The native aortic valve was preserved.

**Figure 2:**
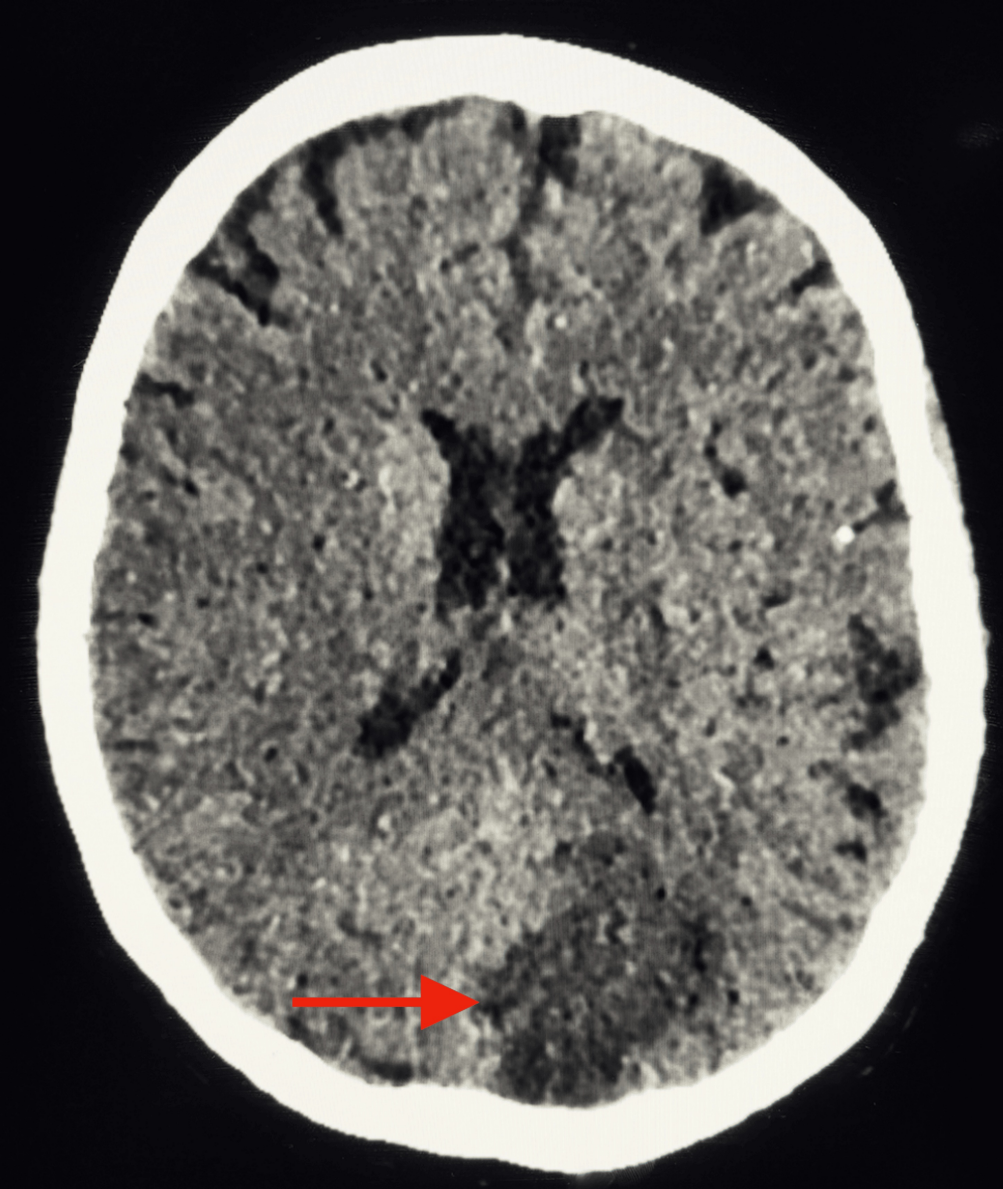
Transverse section of a brain CT scan demonstrating postoperative ischemic lesions (red arrow).

In the immediate postoperative period, following extubation, the patient developed right homonymous hemianopia and right hemiparesis. Brain CT showed multiple established ischemic lesions in the occipital lobes and the head of the caudate nucleus (Figure 2).

The patient’s ICU course was marked by partial recovery of neurological deficits. She was discharged from the ICU on postoperative day 4 and from the hospital on day 16.

## Discussion

Ascending aortic aneurysms exceeding 5.5 cm warrant surgical intervention due to the increased risk of rupture and dissection [1,6]. Giant aneurysms (>10 cm) are exceptionally rare and present significant multidisciplinary management challenges [4,5]. Compression of adjacent structures by such large aneurysms is well documented, particularly affecting the upper airways. This can manifest as chronic cough, dyspnea, stridor, or even dysphonia [5]. In our case, intraoperative bronchoscopy confirmed collapse of the left main bronchus, explaining the patient’s atypical respiratory symptoms.

Chronic cough, defined as lasting more than eight weeks, is a common reason for medical consultation. Frequent causes include asthma, gastroesophageal reflux, and upper airway cough syndrome [7]. However, compressive mediastinal etiologies should always be considered when clinical features such as a cardiac murmur, abnormal chest imaging, or unexplained dyspnea are present. Chest CT is the gold standard for evaluating mediastinal masses and thoracic aortic aneurysms [8]. In this case, diagnosis was achieved through CT, following several months of diagnostic delay, highlighting the critical role of early imaging in persistent unexplained symptoms.

Surgical management of giant aneurysms requires meticulous planning, especially in cases of airway compression. The originality of this report lies in the use of pre-induction CPB cannulation as a precautionary strategy against intubation failure, an approach not previously documented in the literature. Early postoperative neurological complications are a major concern following ascending aorta and arch surgery. The incidence of stroke following ascending aortic surgery is approximately 7% [9]. Identified risk factors include advanced age, cerebrovascular atherosclerosis, prior stroke, and prolonged or complex circulatory arrest [10,11]. Cerebral ischemic lesions following aortic surgery commonly affect the frontal, parietal, occipital lobes, and basal ganglia, as observed in our patient [12]. These complications are attributed to aortic manipulation, air or atheromatous embolism, and prolonged circulatory arrest.

To mitigate the risk of cerebral ischemia, various intraoperative neuroprotective strategies are recommended. Deep hypothermia combined with selective cerebral perfusion has been shown to significantly reduce stroke incidence [11]. Nevertheless, neurological risks remain particularly high in complex aortic arch surgeries. This case underscores the diagnostic and therapeutic complexity of giant ascending aortic aneurysms and highlights the need for a multidisciplinary approach and thorough planning to reduce the risk of severe complications.

## Conclusions

This clinical case emphasizes the need to systematically consider mediastinal compressive causes in the differential diagnosis of chronic unexplained cough, particularly in the presence of evocative clinical signs. Chest CT plays a key role in identifying potentially life-threatening conditions such as giant ascending aortic aneurysms. The surgical management of these aneurysms, especially when airway compression is present, must be anticipatory and multidisciplinary, incorporating neuroprotective strategies to minimize postoperative neurological complications.
